# Long-Term Results of Single- and Multi-Incision Minimally Invasive Esophagectomy for Esophageal Cancer: Experience of 348 Cases

**DOI:** 10.3390/biomedicines13071523

**Published:** 2025-06-21

**Authors:** Yung-Hsin Chen, Pei-Ming Huang, Ke-Cheng Chen, Jang-Ming Lee

**Affiliations:** Division of Thoracic Surgery, Department of Surgery, National Taiwan University Hospital and National Taiwan University College of Medicine, Taipei 100233, Taiwan

**Keywords:** minimally invasive esophagectomy, single-port, esophageal cancer survival

## Abstract

**Importance:** While minimally invasive esophagectomy is currently accepted as an effective treatment for patients with esophageal cancer, the long-term survival outcomes of single-incision minimally invasive esophagectomy in these patients are still unknown, particularly when compared to those of the more invasive multi-incision minimally invasive esophagectomy. **Objective:** To determine the long-term oncological outcomes of single-incision minimally invasive esophagectomy in patients with esophageal cancer and to compare these outcomes with those of multi-incision minimally invasive esophagectomy. **Design:** This was a prospective, randomized, and propensity score-matched study wherein we analyzed patients who underwent treatment from February 2005 to May 2022. **Setting:** Our study was carried out by a single surgical team in a tertiary medical center. **Participants:** We analyzed 348 patients with esophageal cancer who underwent single-incision minimally invasive esophagectomy and 469 who underwent multi-incision minimally invasive esophagectomy. **Main Outcomes and Measures:** We aimed to determine the long-term survival outcomes of single-incision minimally invasive esophagectomy and compare these to those of multi-incision minimally invasive esophagectomy in our study population, and further conducted a propensity score-matching (n = 251 in each arm) study. **Results:** The disease progression-free (DFS) and overall survival (OS) rates of patients who underwent single-incision minimally invasive esophagectomy (SIMIE) was significantly better than that of those who underwent by multi-incision minimally invasive esophagectomy (MIMIE) (*p* = 0.024 for OS and *p* = 0.027 for PFS). This trend of difference was observed in the subsequent propensity-score matching analysis (*p* = 0.009 and 0.016 for OS and PFS, respectively). **Conclusions and Relevance:** The single-incision technique applied in minimally invasive esophagectomy to treat esophageal cancer is feasible without compromising the patient’s long-term oncological outcome, as opposed to that applied using multi-incision minimally invasive esophagectomy.

## 1. Introduction

Esophageal cancer has an increasing impact on the global health burden, with a rapidly rising incidence rate in certain geographic areas [1,2]. Esophagectomy with or without chemotherapy or radiotherapy remains the mainstay treatment for resectable esophageal cancer. Minimally invasive esophagectomy (MIE), including thoracoscopic esophagectomy and laparoscopic gastric mobilization, has been accepted as an effective surgical approach to treat esophageal cancer, with oncological outcomes equivalent to those of traditional open esophagectomy but with fewer pulmonary complications [3,4,5].

Further enhancement of the effect of minimally invasive surgery through reducing ports was proposed until only a single port was used during surgery [6,7,8]. The single-port minimally invasive surgery, defined by only one surgical incision being created during laparoscopic or thoracoscopic surgery, was first adopted for simple surgical procedures, such as appendectomy and cholecystectomy, before gradually being applied to more complex resection procedures, including colectomy, gastrectomy, and even sleeve pulmonary lobectomy [9,10,11,12]. A meta-analysis of the treatment of lung cancer showed that single-port video-assisted thoracic surgery (VATS), as compared to multi-port VATS, can significantly reduce postoperative wound pain while providing an equivalent perioperative outcome for the patients [13]. Previously, we also demonstrated that single-incision MIE (SIMIE) can be feasibly used to treat patients with esophageal cancer and achieve comparative perioperative outcomes with reduced postoperative pain compared to the outcomes achieved using multi-incisional approaches (MIMIE) [14,15]. Our hypothesis is that an equivalent oncological and survival outcome can be achieved through SIMIE to that obtained using standard MIMIE. We therefore aimed to compare the overall survival (OS) and progression-free survival (PFS) of patients who underwent single-incision MIE and those who underwent multi-incision MIE through a propensity score-matching study (PSM).

## 2. Patients and Methods

### 2.1. Patient Selection

We enrolled 817 patients with esophageal cancer who underwent SIMIE (348) and MIMIE (469) at our institute from February 2005 to May 2022. This study cohort included those patients who were enrolled in a prospective randomized trial for the comparison of SIMIE and MIMIE from August 2018 to December 2020 (n = 50 in each arm) (NTC03646110). This study was approved by the Institutional Review Board (IRB) of the National Taiwan University Hospital (IRB: 202401178RINE). The requirement for informed consent was waived owing to the retrospective nature of the study.

A preoperative staging of esophageal cancer was based on computed tomography scans of the brain, neck, chest, and abdomen; positron emission tomography; endoscopic ultrasound; bronchoscopy examination; and upper gastrointestinal barium study. Neoadjuvant concurrent chemoradiation (CCRT; neoCCRT) was provided for the patients with clinical staging of T3/4 or N ≥ 1 4–8 weeks before surgery.

### 2.2. Surgical Techniques

We started SIMIE and MIMIE (four ports used in the thoracic and abdominal phases, respectively) in our institute, as previously described in the literature [14,15], with patients placed in a left decubitus position during the thoracic phase and in a supine position during the abdominal phase. For SIMIE, as shown in Videos S1 and S2, the operator stood on the dorsal side of the patient, while the scopist or assistant stood on the ventral side. We made a 3–4 cm incision through the 4th–5th intercostal space on the midaxillary line in the thoracic phase in both McKeown (cervical anastomosis) and Ivor Lewis (intrathoracic anastomosis) esophagectomies. A three-field lymph node dissection of the thoracic, abdominal, and cervical areas was performed for patients with esophageal squamous cell carcinoma, and two-field dissection was performed for those with esophageal adenocarcinoma without cervical dissection. Video S1 demonstrates the procedures of single-incision thoracoscopic esophagectomy and intra-thoracic lymphadenectomy. These procedures were performed in the thoracic phase of minimally invasive Ivor Lewis esophagectomy. The procedure was McKeown esophagectomy and intrathoracic esophagogastrostomy, carried out using a DST Orvil device circular stapler device (Medtronic Co., MN, USA). For the single-incision laparoscopic procedure, we made a 3 cm periumbilical incision with a surgical wound protector (GelPoint; Applied Medical, Rancho Santa Margarita, CA, USA; GlovePort; Nelis Corporation, Bucheon, Republic of Korea), through which two or three surgical instruments and a 10 mm camera were inserted during the procedure. The liver was retracted with a self-retaining stitch that was passed through the diaphragmatic hiatus and tightened extracorporeally. We performed a feeding jejunostomy for all, unless the patient had already undergone the procedure prior to CCRT. A 10 mm 300 three-dimensional (3D) scope (Karl Storz SE, Tuttlingen, Germany) was used during the laparoscopic and thoracoscopic procedures. For Ivor Lewis esophagectomy, intrathoracic anastomosis was performed using a circular stapler (OrVil; Covidien, Dublin, Ireland) (Video S2).

### 2.3. Statistical Analysis

For comparisons of the baseline clinicopathological characteristics, perioperative outcomes, and oncological outcomes of SIMIE and MIMIE, Student’s *t*-test was used for continuous variables, while Fisher’s exact test and Pearson’s χ2 test were used for categorical variables. OS and PFS curves were estimated using the Kaplan–Meier method.

Propensity score-matching was performed using a 1:1 nearest neighbor-matching algorithm without replacement, with a caliper of 0.25 standard deviations. Propensity score-matching was performed based on the following variables: age, sex, Ivor Lewis or McKeown esophagectomy, tumor pathology, neoCCRT status, and pathological staging.

The analyses were performed using SAS 9.4 (SAS Inc., Cary, NC, USA). A two-sided *p* < 0.05 indicated statistical significance.

## 3. Results

**Figure 1:**
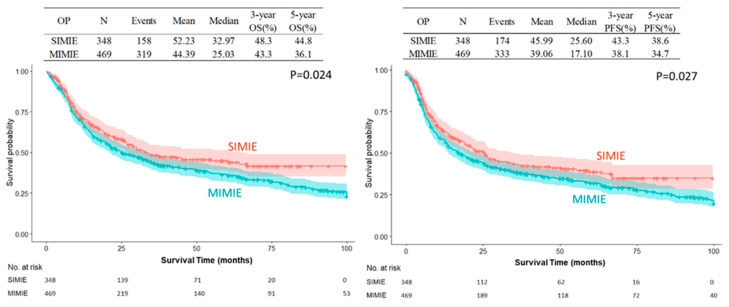
Kaplan–Meier survival curves of SIMIE and MIMIE OS and PFS in the unmatched cohort. The SIMIE group had significantly higher OS and PFS compared to the MIMIE group (*p* = 0.024 and 0.027, respectively). MIMIE, multi-incision minimally invasive esophagectomy; OS, overall survival; PFS, progression-free survival; SIMIE, single-incision minimally invasive esophagectomy.

**Figure 2:**
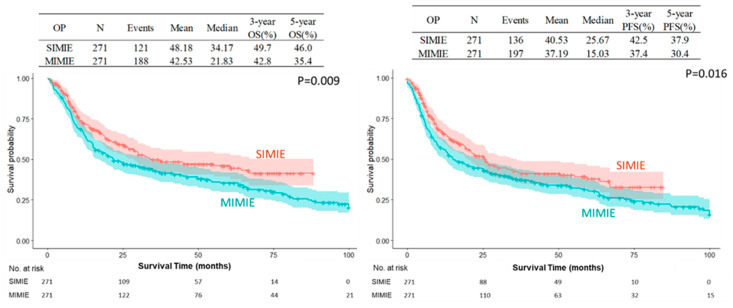
Kaplan–Meier survival cures of SIMIE and MIMIE OS and PFS in propensity score-matched cohort. The SIMIE group had significantly higher OS and PFS compared to the MIMIE group (*p* = 0.009 and 0.016, respectively). MIMIE, multi-incision minimally invasive esophagectomy; OS, overall survival; PFS, progression-free survival; SIMIE, single-incision minimally invasive esophagectomy.

The SIMIE group was associated with more McKeown esophagectomy (247 of 348 [71.0%] vs. 285 of 469 [60.8%], *p* = 0.0025) and a significantly higher incidence of neoCCRT and stage II and III disease; this became balanced after propensity-matching between the two groups of patients, which showed no difference in the distribution of age, sex, Ivor Lewis or McKeown esophagectomy, tumor pathology, neoCCRT status, and pathological staging, as shown in Table 1. The perioperative outcomes are shown in Table 2. There were no significant differences in the overall or individual postoperative complication rates between the SIMIE and MIMIE groups. Table 2 shows the oncological outcomes of both groups. More lymph nodes were retrieved in the SIMIE group in the unmatched and propensity score-matched cohorts (*p* < 0.05). Significantly better OS and PFS rates of 3 and 5 y were found after surgery in the unmatched and propensity score-matched SIMIE cohorts than in MIMIE (*p* = 0.0244 for OS and 0.0305 for PFS in the unmatched cohort; *p* = 0.008 for OS and 0.02 for PFS in the propensity score-matched cohort, respectively) (Table 2). Figure 1 and Figure 2 demonstrate the Kaplan–Meier survival curves for SIMIE and MIMIE. A better OS and PFS can be observed in the SIMIE cohort than in the MIMIE cohort in the unmatched (Figure 1) and propensity score-matched cohorts (Figure 2) (*p* < 0.05, respectively).

## 4. Discussion

Our results demonstrate that SIMIE can provide acceptable oncological long-term outcomes compared to MIMIE. This result was further verified by PRS and PSM. From August 2018 to December 2020, a prospective randomized study was carried out comparing the oncological and perioperative outcomes of SIMIE and MIMIE (NTC03646110). Fifty patients were enrolled in each arm, who showed no statistical difference in perioperative complication and mid-term survival outcomes when looking at 3-year overall and progression-free survival (56.0%, 53.4% for OS and 50.0%, 46.0% for PFS of the SIMIE and MIMIE, respectively). It seems that SIMIE does not compromise the surgical results when compared to MIE. We proceeded to perform SIMIE to treat most patients of esophageal cancer in our institute thereafter. The current series was collected from our patient cohort, most of whom presented with locally advanced disease upon diagnosis, in which 73% of the patients received neoadjuvant chemoradiation before surgery, and all of the patients with squamous cell carcinoma received three-field lymphadenectomy, irrespective of whether the McKeown or Ivor Lewis approaches were used. To further minimize selection bias, PSM was conducted to compare the two surgical approaches. There was no significant difference in postoperative 30 d mortality and major postoperative complications between the SIMIE and MIMIE groups in the PSM analyses. However, there were differences in OS and PFS three and five years after esophagectomy; these were significantly better in SIMIE patients overall and following PSM, as shown in Figure 1 and Figure 2, respectively.

To facilitate the effectiveness and safety of tissue dissection during surgery, several technical issues must be addressed when using the single-incision approach in minimally invasive surgeries. The deep-seated location of the esophagus makes the working space of instruments extremely narrow, making it difficult to clearly visualize the target areas. Several technical modifications were made to the SIMIE procedure based on our experience. The patients were kept in the left lateral position with the surgeon standing on the right side of the patient, and a 3–4 cm incision was made with the surgeon’s right hand along the midaxillary line at the 4th–5th intercostal space to facilitate tissue dissection along the whole thoracic esophagus, especially the upper mediastinum. An incision was made slightly anterior to the esophagus to prevent obstruction of the esophagus during lymph node dissection into the contralateral mediastinum. For effective retraction of the collapsed lung, various pleural tenting stitches on the mediastinal pleura were made, without the need for an additional assistant port [14,15]. Similarly, the liver was also retracted using a tenting technique through stitch anchoring on the hiatal crus and fixed extracorporeally [4]. This dissection of the periesophageal area in the thoracic and abdominal region was performed with the need for retraction from the assistant, which required the creation of a surgical port. In addition to the technical considerations, a 3D imaging system was also helpful in ensuring the quality and safety of tissue dissection during the procedure. Previously, the 3D imaging system was found to be helpful in reducing surgical blood loss and shortening the drainage duration in VATS [16]. Shen et al. also found that the use of a 3D imaging system during VATS segmentectomy can facilitate a reduction in the number of port creations, blood loss, and length of stay [17]. With the aid of this imaging system, the operator can identify the target without the need to maintain a close distance during the procedure, and sufficient space can be more easily provided for the movement of surgical instruments and for precise dissection during single-incision surgery. We found that these technical modifications were useful for the surgical treatment of esophageal cancer, especially when completing a radical lymph node dissection, and helped to avoid issue damage. Owing to the high incidence of lymph node metastasis along the bilateral upper mediastinum [18,19], it is important to ensure complete lymph node dissection in these areas during esophagectomy. Radical lymph node dissection was achieved, with an acceptable complication rate of vocal cord palsy (4.3%) in our series of SIMIE, without compromised results regarding the number of dissected lymph nodes, when compared to multi-port MIE [14,15].

To ensure sufficient proximal margins after surgery, the patients underwent endoscopic clip localization at the proximal end of the tumor. A frozen pathological examination was also performed if the proximal margin after resection was less than 1 cm. However, most of the patients presented with locally advanced disease status, with 70% having a greater indication for neoadjuvant chemoradiation. This could mainly be attributed to the fact that approximately 10% of patients in both groups underwent R1 or R2 resection in terms of the circumferential margin, which was compatible with the observations of a previous study [20].

Our study presents the evolution of the minimally invasive surgical technique to treat esophageal cancer, from open, muti-portal, reduced-port MIE to single-port MIE. Mastering SIMIE requires time for the maturation of surgical skills and to train the surgical team using the principles and experience obtained from performing MIMIE. After the surgical team became familiar with the setting and procedures of SIMIE, we performed a PRS to ensure the safety and effectiveness of SIMIE compared with MIMIE. The perioperative and oncological outcomes of SIMIE were not inferior compared to those of MIMIE. Therefore, SIMIE was performed as a routine procedure to treat patients with esophageal cancer. In this series, we analyzed the entire cohort of patients with esophageal cancer who underwent MIE to further clarify the long-term survival outcome of SIMIE. PSM was used to minimize bias in patient selection in the MIMIE and SIMIE groups. After matching for age, sex, types of esophagectomy, pathological cell types, pathological staging, and CCRT, 271 patients were recruited from each group for propensity score-matching. No significant difference was observed between the two groups in terms of surgical complication rates. The operation time was significantly longer for MIMIE, whereas the hospital and intensive care unit lengths of stay were similar. However, OS and PFS were significantly better in the SIMIE group than in the whole and propensity-matched patient cohorts. The reasons for the difference in survival between the SIMIE and MIMIE groups are unclear. However, there are several possible explanations for our observation. First of all, the number of retrieved lymph nodes were significantly higher in the whole and PMS cohorts. Previous studies also demonstrated that different surgical approaches, including open, multi-portal, and uni-portal VATS, can induce different degrees of postoperative inflammatory response and muscle-wasting [21,22], which can impact the survival of the patient after esophagectomy [23,24,25,26]. Nonetheless, it is obvious that the long-term survival outcome was not compromised by the use of the single-port technique in MIE procedures in our series. Therefore, SIMIE is gradually being applied to more complex surgical procedures for the treatment of patients with esophageal cancer. Twenty-three patients underwent total laryngopharyngoesophagectomy and permanent tracheostomy, and fifty-one patients with aortic invasion (T4b) tumors were resected following thoracic endovascular aortic repair and CCRT [27]. With the gradually increasing popularity of the single-port surgical technique in treating thoracic diseases, SIMIE has also been studied by several authors in Asia and Europe, who achieved comparable perioperative and oncological outcomes to those achieved following MIMIE or open surgery [28,29,30,31,32]. We believe our results could provide stimulate the refinement of MIE for the treatment of esophageal cancer through the use of various cutting-edge technologies, including artificial intelligence and robotic surgery.

There are certain limitations to our study, primarily due to selection bias attributed to the different time periods of the development of these two approaches and the use of different surgical techniques, although both followed the same surgical principles for lymph node dissection, tumor resection, and esophageal reconstruction, and all of the patients followed similar treatment guidelines before and after surgery. All of the patients went through a similar multi-modality treatment before and after surgery in our institute. Although all possible confounders were balanced in PSM, further multi-institutional studies are needed to confirm our findings.

In conclusion, SIMIE might be feasible and effective for patients with esophageal cancer once radical lymph node dissection can be carried out using this approach. Its long-term oncological outcomes are comparable with those of MIMIE.

## Figures and Tables

**Table 1:** Clinicopathological characteristics of SIMIE and MIMIE in unmatched and propensity score-matching (nearest neighbor matching) cohorts.

	No. (%)					
	Unmatched cohort (n = 817)			Propensity score matching (n = 542)		
**Characteristic**	SIMIE	MIMIE	*p*-value	SIMIE	MIMIE	*p*-value
	(n = 348)	(n = 469)		(n = 271)	(n = 271)	
**Age, median (IQR), y**	58.5 (52.0–65.0)	58.0 (52.0–65.0)	0.9828	58.0 (52.0–65.0)	58.0 (52.0–65.0)	0.7719
**Sex**			0.0760			1.0000
Male	314 (90.2)	439 (93.6)		263 (97.0)	263 (97.0)	
Female	34 (9.8)	30 (6.4)		8 (3.0)	8 (3.0)	
**Lung function, median (IQR), %**						
FVC	106.8 (95.0–117.6)	103.8 (92.0–113.9)	0.2237	106.5 (94.3–117.1)	102.7 (91.5–113.9)	0.1242
FEV1	102.4 (91.3–113.6)	101.3 (90.5–111.4)	0.8993	102.0 (91.6–113.4)	100.8 (90.1–112.2)	0.8539
**Pre-op Alb level, median (IQR), g/dL**	4.2 (3.9–4.4)	4.2 (3.8–4.4)	0.2267	4.2 (3.9–4.4)	4.2 (3.9–4.4)	0.2062
**Pathology type**			0.6164			1.0000
SCC	314 (92.1)	422 (90.7)		264 (97.4)	264 (97.4)	
Adenocarcinoma	23 (6.7)	36 (7.7)		7 (2.6)	7 (2.6)	
Others	4 (1.2)	8 (1.7)				
**Pathological staging**			0.2292			0.5492
0	4 (1.2)	4 (0.9)		4 (1.5)	1 (0.4)	
I	175 (50.4)	224 (48.4)		144 (53.1)	139 (51.3)	
II	29 (8.4)	60 (13.0)		27 (10.0)	35 (12.9)	
III	111 (32.0)	148 (32.0)		76 (28.0)	78 (28.8)	
IV	28 (8.1)	27 (5.8)		20 (7.4)	18 (6.6)	
**Neoadjuvant CCRT**			0.1247			1.0000
**+**	254 (73.0)	319 (68.0)		208 (76.8)	208 (76.8)	
**–**	94 (27.0)	150 (32.0)		63 (23.3)	63 (23.3)	
**Operation method**			0.0025			1.0000
McKeown	247 (71.0)	285 (60.8)		199 (73.4)	199 (73.4)	
Ivor Lewis	101 (29.0)	184 (39.2)		72 (26.6)	72 (26.6)	

Alb, albumin; CCRT, concurrent chemoradiotherapy; FEV, forced expiratory volume; FVC, forced vital capacity; IQR; interquartile range; MIMIE, multi-incision minimally invasive esophagectomy; SCC, squamous cell carcinoma; SIMIE, single-incision minimally invasive esophagectomy.

**Table 2:** Perioperative and oncological outcomes of SIMIE and MIMIE in unmatched and propensity score-matching (nearest neighbor matching) cohorts.

	No. (%)					
	Unmatched cohort (n = 817)			1:1 Propensity score matching (n = 542)		
**Characteristic**	SIMIE	MIMIE	*p* value	SIMIE	MIMIE	*p* value
	(n = 348)	(n = 469)		(n = 271)	(n = 271)	
**Postoperative overall complication rate (%)**	65 (18.7)	70 (14.9)	0.1532	50 (18.5)	36 (13.3)	0.0998
Pulmonary (%)	10 (2.9)	16 (3.4)	0.6649	7 (2.6)	9 (3.3)	0.6118
Leakage (%)	14 (4.0)	9 (1.9)	0.0722	12 (4.4)	5 (1.9)	0.0845
**30-d Mortality**						
(%)	5 (1.4)	14 (3.0)	0.1465	3 (1.1)	7 (2.6)	0.2017
**LNs retrieved**	43 (29–57)	36 (21–49)	<0.0001	44 (32–58)	36 (23–49)	<0.0001
**Resection margin**			0.3494			0.8838
R0	298 (89.0)	346 (91.0)		231 (87.8)	203 (88.3)	
R1 or R2	37 (11.0)	34 (9.0)		32 (12.2)	27 (11.7)	
**Overall survival**			0.0244			0.0088
3 y	0.4832	0.4335		0.4975	0.4284	
5 y	0.4484	0.3611		0.4597	0.3537	
**Progression** **-free survival**			0.0305			0.0207
3 y	0.4281	0.3845		0.4246	0.3798	
5 y	0.3863	0.3219		0.3787	0.3086	

LN, lymph node; MIMIE, multi-incision minimally invasive esophagectomy; SIMIE, single-incision minimally invasive esophagectomy.

## Data Availability

The authors confirm that the data generated and analyzed during this study and the raw data are available from the corresponding author, upon reasonable request.

## Supplementary Materials

The following supporting information can be downloaded at: https://www.mdpi.com/article/10.3390/biomedicines13071523/s1, Video S1: Single-incision thoracoscopic esophagectomy and intra-thoracic lymphadenectomy (Ivor Lewis esophagectomy); Video S2: Single-incision laparoscopic gastric mobilization and intra-abdominal lymphadenectomy.
